# Pharmacokinetics of mycophenolic acid in plasma and peripheral blood mononuclear cells and its relationship with activity of inosine monophosphate dehydrogenase in Chinese adult kidney allograft recipients

**DOI:** 10.3389/fphar.2025.1712932

**Published:** 2025-11-19

**Authors:** Yun-Zhu Lu, Xiao-Ling Lu, Jia-Qian Lu, Kun Shao, Hui-Min An, Pei-Jun Zhou, Hao-Qiang Shi, Bing Chen

**Affiliations:** 1 Department of Pharmacy, Ruijin Hospital, Shanghai Jiao Tong University School of Medicine, Shanghai, China; 2 Center for Organ Transplantation, Ruijin Hospital, Shanghai Jiao Tong University School of Medicine, Shanghai, China

**Keywords:** mycophenolic acid, pharmacokinetics, pharmacodynamics, peripheral blood mononuclear cell, inosine monophosphate dehydrogenase

## Abstract

**Aims:**

We aimed to study the pharmacokinetics (PK) of mycophenolic acid (MPA) in plasma and peripheral blood mononuclear cells (PBMCs) and the relationship of MPA in plasma and PBMC with activity of inosine monophosphate dehydrogenase (IMPDH) in Chinese kidney allograft recipients.

**Methods:**

Plasma and PBMC samples were collected 0–12 h after administration of mycophenolate mofetil (MMF) or enteric-coated mycophenolate sodium (EC-MPS) 2 weeks after initiation of immunosuppressive therapy. MPA concentrations in plasma and PBMCs and IMPDH activity were determined using liquid chromatography–mass spectrometry. The PK and pharmacodynamic parameters was estimated. The relationship between plasma and PBMC MPA levels and IMPDH activity was determined using the inhibitory E_max_ model.

**Results:**

Totally 24 patients receiving MMF and 19 patients receiving EC-MPS were included in the study. A significant positive correlation was observed between the C_0_ (r = 0.535, P = 0.007), C_max_ (r = 0.538, P = 0.007), and AUC_0–12h_ (r = 0.472, P = 0.02) of plasma and PBMC MPA in patients who received MMF. IMPDH activity exhibited a negative correlation with MPA plasma and PBMC concentrations. Significant relationship was observed between MPA exposure and IMPDH activity in PBMC (r = 0.398, P = 0.01). The IC_50_ of plasma and PBMC MPA on IMPDH activity were 2.76 μg/mL and 0.023 ng/10^6^ cells for MMF, 3.34 μg/mL and 0.052 ng/10^6^ cells for EC-MPS, respectively.

**Conclusion:**

Measurement of MPA concentration and IMPDH activity in PBMCs serves as a complementary method to routine clinical therapeutic drug monitoring, which provide basis for the individual therapy of MPA in Chinese kidney allograft recipients.

## 1 Introduction

Kidney transplantation is a common and effective treatment for end-stage kidney disease. Progress in therapeutic immunosuppression has considerably reduced the incidence of transplant rejection (Alasfar et al., 2023; Pilch et al., 2021). Mycophenolic acid (MPA) is the active form of mycophenolate mofetil (MMF) and enteric-coated mycophenolate sodium (EC-MPS), which are used in combination with calcineurin inhibitors, including tacrolimus (TAC) or cyclosporine A (CsA), and corticosteroids for immunosuppressive therapy following solid organ transplantation (Rong et al., 2021; Oliveras et al., 2024; Jung et al., 2025; Al-Khouja et al., 2024), hematopoietic stem cell transplantation (Zhang and Chow, 2017), or in autoimmune disease treatment (Abd Rahman et al., 2013).

MMF and EC-MPS are usually administered at a recommended dose of 1000 mg bid and 720 mg bid, respectively. While both formulation deliver the active moiety MPA, their pharmacokinetic (PK) profiles differ primarily due to formulation. EC-MPS is designed to resist dissolution in the stomach, thereby delaying MPA release until the tablet reaches the small intestine. This mechanism aims to reduce upper GI adverse effects but can result in a delayed T_max_ (the time at which the C_max_ is reached) and higher inter-individual variability in absorption compared to MMF. However, the MPA exposure (expressed as area under the concentration-time curve, AUC) is comparable between different formulations (Graff et al., 2016). Earlier studies revealed that MPA exposure is associated with immunosuppressive as well as adverse effects (van Gelder et al., 2006; Bergan et al., 2021; Milesi et al., 2024). Therapeutic drug monitoring of MPA is helpful for patients receiving MMF or EC-MPS, and AUC within the range of 30–60 μg·h⋅mL^−1^ in kidney transplant recipients is accepted as the target range (Meziyerh et al., 2023; Kuypers et al., 2010). There are significant inter- and intra-individual variations in MPA PK, which are attributed to various factors, including genetic polymorphism of metabolic enzymes and transporters as well as use of concomitant drugs (Ferreira et al., 2020; Na Takuathung et al., 2021; Chen et al., 2019; Yap et al., 2020; Jiang and Hu, 2021). As MPA exerts its effects mainly within lymphocytes, the intracellular MPA level, rather than its plasma level, is assumed to be more closely associated with efficacy. The equilibrium between influx and efflux mechanisms could influence the intracellular MPA PK in lymphocytes, thereby impacting its therapeutic effect. However, studies on intracellular MPA PK remain relatively scarce (Nguyen et al., 2013; Md Dom et al., 2018).

MPA exerts its effects by selectively, non-competitively, and reversibly inhibiting inosine monophosphate dehydrogenase (IMPDH), which in turn inhibits lymphocyte proliferation (Ferreira et al., 2020; Staatz and Tett, 2014). IMPDH catalyzes the conversion of nicotinamide adenine dinucleotide (NAD)-dependent oxidation of inosine-5′-monophosphate to xanthosine-5′- monophosphate (XMP) and is proposed as a potential pharmacodynamic (PD) index for optimizing MPA therapy (Weißbarth et al., 2020). Large inter-patient variability is reported to exist in both baseline IMPDH activity and the time required for complete recovery of enzyme activity after MPA administration (Sobiak et al., 2022; Glander et al., 2021). IMPDH is highly expressed in lymphocytes. Therefore, measuring IMPDH activity within PBMCs provides a more direct and pharmacodynamically relevant assessment of MPA effect (Klaasen et al., 2020; Winnicki et al., 2022). Elucidating the relationship between IMPDH biological activity and MPA PK in plasma or PBMCs of kidney allograft recipients is valuable.

In this study, we aimed to evaluate the correlation between plasma and intra-PBMC MPA PK in patients undergoing treatment with MMF or EC-MPS. Furthermore, we aimed to assess the PK–PD relationship in plasma and PBMC concerning IMPDH inhibition. This can help improve our understanding of the role of IMPDH as a clinical biomarker for assessing MPA efficacy.

## 2 Methods

### 2.1 Patients and immunosuppression protocol

In this retrospective study, data from 43 patients who underwent kidney transplantation between 2020 and 2024 in Ruijin Hospital were included. The inclusion criteria for patients were as follows: (1) early-stage primary kidney transplant recipients receiving from a standard kidney donor; (2) those with no immunosuppressant administered before transplant surgery; (3) those on postoperative immunosuppressive drug maintenance regimen, namely, MMF or EC-MPS in combination with CsA or TAC and glucocorticoids; and (4) those with no dosage adjustment of MMF or EC-MPS within the first week after the operation.

The exclusion criteria for patients included: (1) those with combined organ transplantation; (2) those with panel-reactive antibody positivity; (3) those with allergy or intolerance to MMF, EC-MPS, CsA, TAC, or glucocorticoids; (4) those with history of serious adverse reactions (especially gastrointestinal reactions) related to immunosuppressive drugs; and (5) pregnant or lactating women.

A dose of 1000 mg MMF (Cellcept, Roche) or 720 mg EC-MPS (Myfortic, Novartis Pharma) was administered within 6 h before kidney transplantation. The same dose was administered every 12 h after transplantation. Thereafter, doses were adjusted according to MPA concentration, clinical efficacy, and toxicity. A dose of 7 mg·kg^−1^·day^−1^ CsA (Sandimmun-Neoral, Novartis Pharma) was initially administered twice daily from the third day post-transplantation. Subsequently, doses were adjusted to achieve a target C_0_ of 200–250 ng·mL^−1^ in the first month post-transplantation and 150–200 ng·mL^−1^ thereafter. TAC (Prograf, Astellas) was initially administrated at 0.1 mg·kg^−1^·day^−1^ twice daily and then adjusted to a C_0_ of 10–13 ng·mL^−1^ in the first month and 5–9 ng·mL^−1^ thereafter. Methylprednisolone (Pfizer, Puurs) was intravenously administered during surgery and progressively tapered. The patients were maintained on a daily oral prednisone dose of 5–10 mg after the first month post-transplantation.

### 2.2 PK study of MPA

Complete PK profiles were obtained from patients 2 weeks (11–17 days) after the initiation of treatment. Peripheral venous blood samples (5 mL for each time point) were collected in EDTA-anticoagulated tubes before dosing and at 0.5, 1, 1.5, 2, 4, 6, 8, 10, and 12 h after dosing. Thereafter, 1 mL of each blood sample was centrifuged at 1610 *g* for 10 min, and plasma was collected and stored at −80 °C before determination of MPA concentration. For the other samples, PBMCs were isolated from the whole blood. Briefly, 3.5 mL of PBS was added to equal volume of whole blood samples and mixed gently. Thereafter, the mixture was carefully poured into *BD* Falcon *polypropylene tubes* (*BD* Biosciences) containing 3.5 mL Ficoll-Paque Plus solution (GE Healthcare Bio-Sciences AB, Uppsala, Sweden), without mixing. After centrifugation (at 400 × *g*, 40 min, 20 °C), the PBMC layers were collected and further centrifuged at 12,000 *g* for 10 min, and the supernatant was removed. The obtained PBMCs were washed twice (using 6 mL PBS, at 4 °C), resuspended with PBS, and divided into two parts, which were used for the determination of MPA and XMP (index of IMPDH activity), respectively. Before storing at −80 °C, a 10 μL aliquot was used for hemocytometry under a microscope.

### 2.3 Determination of MPA in plasma and PBMC

The liquid chromatography–tandem mass spectrometry (LC–MS/MS) method established in our lab was used to determine MPA levels in plasma (C_p_), and PBMCs (C_PBMC_), with minor modifications (Chen et al., 2020). The MPA standards were provided by Sigma-Aldrich (Lot #124M4002V). The internal standard (IS) MPA-d_3_ was purchased from Sigma-Aldrich (lot #FN08191401; St. Louis, MO, United States). Methanol and acetonitrile (HPLC grade) were purchased from Tedia (Fairfield, United States). Ammoniacal and formic acids were of reagent grade. Ultra-pure water was obtained using a Millipore water-purification system. The protein precipitation method was used to treat the plasma and PBMC samples.

The LC–MS/MS system used in the present study comprised an LC-20AD (Shimadzu, Kyoto, Japan) system and an API4000 triple quadruple mass spectrometer (Applied Biosystems, Singapore), equipped with electrospray ionization (ESI) operating in the positive mode. Chromatographic separation was achieved using a Zorbax C_18_ column (50 × 2.1 mm, particle size: 3.5 μm; Agilent, Lexington, MA). The mobile phase consisted of (A) water containing 2 mM ammonium formate and (B) methanol containing 2 mM ammonium formate. The flow rate was 0.35 mL·min^−1^ with the following elution gradient: 0–0.5 min, solvent B was maintained at 20%; 0.5–1.5 min, solvent B was linearly increased from 20% to 100%; 1.5–5 min, solvent B was maintained at 100%; 5–6 min, solvent B was linearly decreased from 100% to 20%; and 6–7.5 min, solvent B was maintained at 20%. Molecules were detected with a multiple reaction monitoring mode (MRM) according to the following m/z transitions of 321.4→207.1 (MPA) and 324.3→210.0 (MPA–d_3_). The retention times of MPA and MPA–d_3_ were 3.72 min and 3.71 min, respectively. MPA were linear in the range of 0.204-51 μg/mL in plasma and 0.098–39.2 ng/mL in PBMC (*r* ^2^ > 0.99). Intra-day and inter-day coefficients of variation were <15%. Samples were stable under brief room temperature storage and long-term −80 °C storage and three freeze-thaw cycles.

### 2.4 Determination of IMPDH activity

IMPDH in PBMCs can catalyze the conversion of inosine-5′-monophosphate to XMP. Therefore, the activity of IMPDH can be assessed by measuring the concentration of XMP. The detailed procedure is as follows:

1. Enzymatic reaction: 10 μL of Br-AMP (IS, 50 μmol·L^−1^) and 80 μL of incubation buffer (containing 40 mmol·L^−1^ Na^+^, 100 mmol·L^−1^ K^+^, 0.5 mmol·L^−1^ NAD^+^, and 1.0 mmol·L^−1^ inosine-5′-monophosphate) were added to 100 μL of PBMC sample and incubated at 37 °C with shaking at 300 rpm for 150 min. After incubation, 10 μL of 2.5 mol·L^−1^ boric acid was added and vortex-mixed to terminate the reaction.
2. Solid-phase extraction (SPE): We used 200 μL methanol to activate the SPE column (Oasis WAX μElution), after which it was equilibrated with 200 μL water. Thereafter, 200 μL of the enzymatic reaction sample was loaded onto the column, and 200 μL water and 200 μL methanol were used to wash the column. Finally, 200 μL of 5% ammonia-methanol solution was used to elute the XMP from column, and 10 μL of the eluate was used for analysis.
3. LC–MS/MS analysis of XMP: Chromatographic separation was achieved using an Agilent Eclipse XDB-C18 (5 μm, 4.6 × 150 mm). The mobile phase consisted of A: 2 mmol·L^−1^ ammonium formate aqueous solution (pH adjusted to 8 using ammonia) and B: 2 mmol·L^−1^ ammonium formate in methanol. The flow rate was 0.4 mL·min^−1^ with the following elution gradient: 0–2.0 min, solvent B was increased from 2% to 98%; 2.0–4.0 min, solvent B was maintained at 98%; 4.0–4.1 min, solvent B was decreased from 98% to 2%; and 4.1–6.0 min, solvent B was maintained at 2%. Mass spectrometric analysis was performed using ESI-positive mode. Molecules were detected with MRM according to XMP ion transition: m/z 365.2 → 97.3, Br-AMP ion transition: m/z 427.7 → 216.1. IMPDH activity was calculated based on the measured XMP concentration using the following formula:

$$\text{IMPDH activity}=\frac{\text{XMP}\;\text{produced}\times 10^{3}}{\text{Incubation}\;\text{time}\times \text{cell}\;\text{counts}}$$

Unit: pmol·h^−1^·10^−6^ cells.

### 2.5 PK and PD analyses

MPA PK parameters were estimated using a noncompartmental method in the Winnonlin 5.01 computer program (Pharsight Corp, Mountain View, CA). The C_max_ and T_max_ were obtained directly from the concentration–time curve. The t_1/2_ was estimated using least squares regression analysis from the terminal phase of the concentration–time curve using three or four different time points.

The MPA PD parameters derived from IMPDH activity for patients who received MMF and EC-MPS were calculated using the non-compartmental method. The maximum (A_max_) and minimum (A_min_) IMPDH activities were the observed values within the MPA dosing interval. T_min_ was defined as the time to reach the minimum IMPDH activity. The area under the IMPDH activity curve (AEC_0–12_) was calculated using linear trapezoidal rule.

The relationship between MPA plasma or PBMC concentrations and IMPDH were explored with NONMEM® software (version VII) using the first-order conditional estimation method. An inhibitory sigmoid E_max_ model was used to describe the relationship between the concentration of MPA and IMPDH activity based on the following equation:

$$E=E_{0}-\frac{E_{\max}\cdot C^{\gamma }}{{IC}_{50}^{\gamma }+C^{\gamma }}$$

where E is IMPDH activity, calculated as: A/A_0_, E_0_ is the effect before MPA administration (baseline), E_max_ is the maximal possible IMPDH inhibition, C is the MPA concentration in plasma or PBMC, IC_50_ is the MPA concentration required for 50% inhibition, and γ is the Hill coefficient.

### 2.6 Statistical analyses

All data are presented as mean values ±SD. ANOVA was used to compare the differences among the groups. Correlations between MPA exposure (C_0_, C_max_, AUC_0-12_) in plasma and PBMC were assessed using the Pearson test. In addition, correlations between MPA exposure in plasma and PBMC and PD indices (AEC) were also analyzed using the Pearson test.

## 3 Results

### 3.1 Population

The demographic characteristics and clinical data of the 43 patients are shown in Table 1. The mean age of patients at transplantation was 41.8 years (16–69 years), and their mean weight was 64.9 kg (36.0–93.5 kg). A total of 24 and 19 patients received MMF or EC-MPS, respectively. No significant differences were observed in baseline data between patients who received MMF or EC-MPS (*P* > 0.05). The mean dose of MMF was 1534 mg/day, ranging from 1000 to 2000 mg, and the mean dose of EC-MPS was 1129 mg/day, ranging from 720 to 1440 mg/day.

**TABLE 1:** Demographic and clinical data of 43 Chinese kidney transplantation recipients.

Characters (unit)	Mean and SD	
	MMF (n = 24)	EC-MPS (n = 19)
Age (years)	42.4 ± 10.4	41.2 ± 10.1
Sex	Male: 16; Female: 8	Male: 14; Female: 5
Weight (kg)	66.0 ± 13.4	63.7 ± 12.7
Post operation days (days)	12.1 ± 3.37	12.9 ± 3.39
Albumin (g·L^−1^)	36.3 ± 4.86	34.5 ± 4.30
Creatinine clearance rate (L·h^−1^)	67.7 ± 26.3	63.9 ± 24.8
Alanine aminotransferase (U·dL^−1^)	2.13 ± 0.32	2.21 ± 0.29
Aspartate aminotransferase (U·dL^−1^)	1.73 ± 0.14	1.67 ± 0.15
TBIL (μmol·L^−1^)	11.6 ± 4.31	11.3 ± 6.39
Haemoglobin (g·L^−1^)	98.6 ± 19.0	99.8 ± 21.3
White blood cell (WBC) (×10^9^·L^−1^)	7.14 ± 2.54	8.45 ± 3.15
Red blood cell (RBC) (×10^9^·L^−1^)	3.06 ± 0.26	3.04 ± 0.19
Platelet (PLT) (×10^9^·L^−1^)	226 ± 77.4	205 ± 104

### 3.2 MPA concentrations and PK parameters in plasma and PBMCs

**FIGURE 1:**
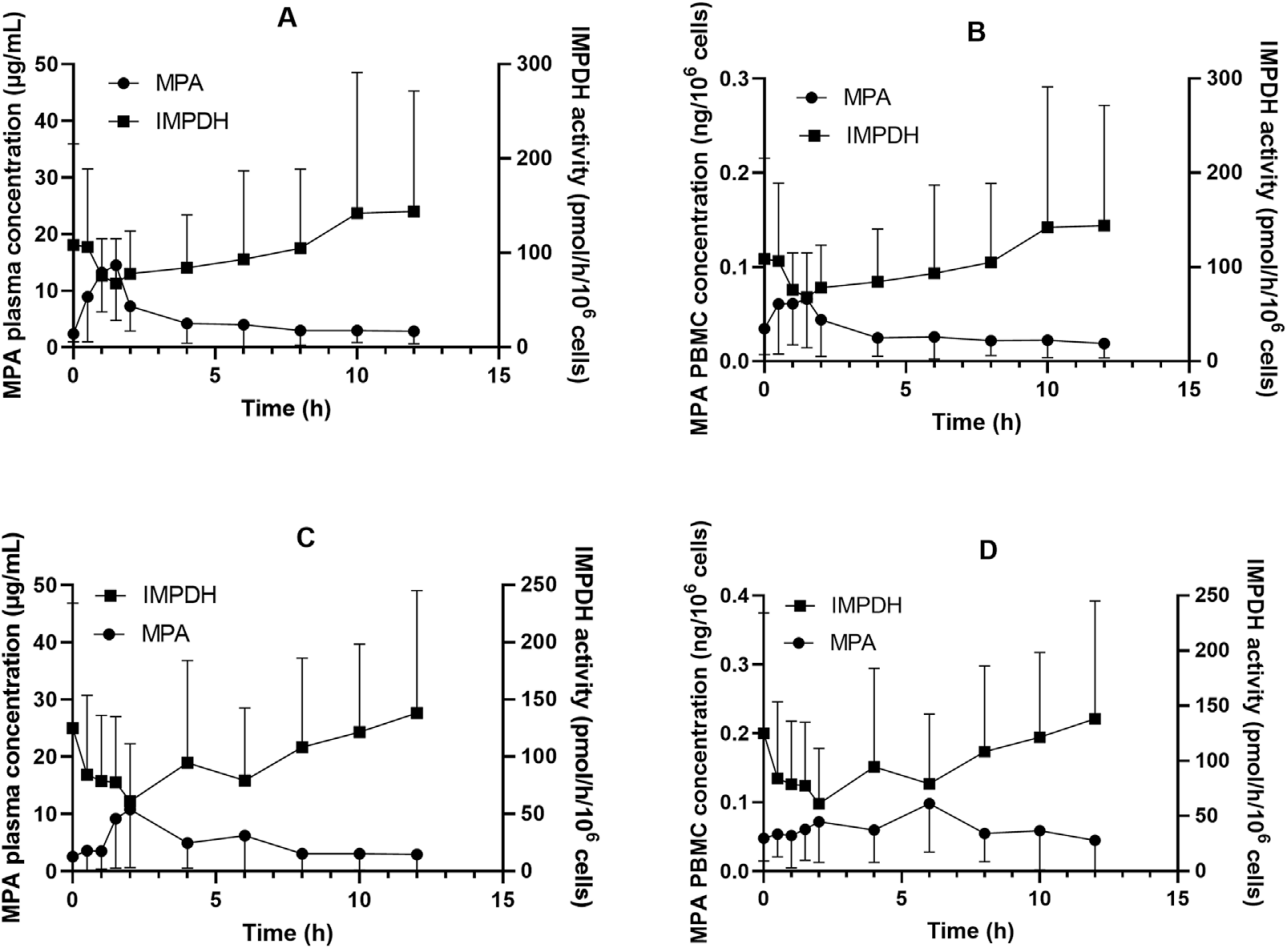
Inosine monophosphate dehydrogenase (IMPDH) activity and mycophenolic acid (MPA) concentrations in plasma and peripheral blood mononuclear cells (PBMC) among Chinese kidney allograft recipients. **(A)** Plasma of patients administered MMF; **(B)** PBMC of patients administered MMF; **(C)** Plasma of patients administered EC-MPS; **(D)** PBMC of patients administered EC-MPS.

The concentration–time curves of MPA in plasma and PBMCs after administration of MMF or EC-MPS are shown in Figure 1. The trends of MPA concentration variation over time were similar in plasma and PBMCs, though inter-individual variability in PBMC concentrations was relatively higher. The PK parameters are presented in Table 2.

**TABLE 2:** MPA pharmacokinetic parameters and IMPDH activity after administration of MMF or EC-MPS in Chinese kidney allograft recipients **(** $\bar{x}$ ± *s* **)**.

		MMF (n = 24)	EC-MPS(n = 19)
C_max_	Plasma (μg·mL^−1^)	18.2 ± 9.28	17.9 ± 9.10
	PBMC (ng·10^−6^cells)	0.10 ± 0.054	0.15 ± 0.052
C_0_	Plasma (μg·mL^−1^)	2.41 ± 1.43	2.57 ± 2.43
	PBMC (ng·10^−6^cells)	0.035 ± 0.028	0.048 ± 0.033
T_max_	Plasma (h)	1.67 ± 1.63	4.03 ± 2.50
	PBMC (h)	1.52 ± 1.22	4.74 ± 3.24
t_1/2_	Plasma (h)	5.20 ± 1.50	4.05 ± 1.69
	PBMC (h)	6.22 ± 2.16	4.77 ± 2.94
AUC_0-12_	Plasma (μg·h·mL^−1^)	39.7 ± 20.9	40.8 ± 19.97
	PBMC (ng·h·10^−6^cells)	0.36 ± 0.22	0.66 ± 0.28
A_min_	(pmol·h^−1^·10^−6^cells)	38.7 ± 21.1	52.9 ± 33.1
A_max_	(pmol·h^−1^·10^−6^cells)	216 ± 146	241 ± 192
T_min_	(h)	2.22 ± 1.63	4.67 ± 2.03
AEC_0-12_	(pmol·10^−6^cells)	1163 ± 592	1146 ± 853

C_max_: maximum concentration; C_0_: trough concentration; T_max_: time of maximum concentration; t_1/2_: half life of elimination; AUC_0-12_: area under the concentration curve; A_min_: minimum activity of IMPDH; A_max_: maximum activity of IMPDH; T_min_: time of minimum activity of IMPDH; AEC_0-12_: area under the effection curve.

**FIGURE 2:**
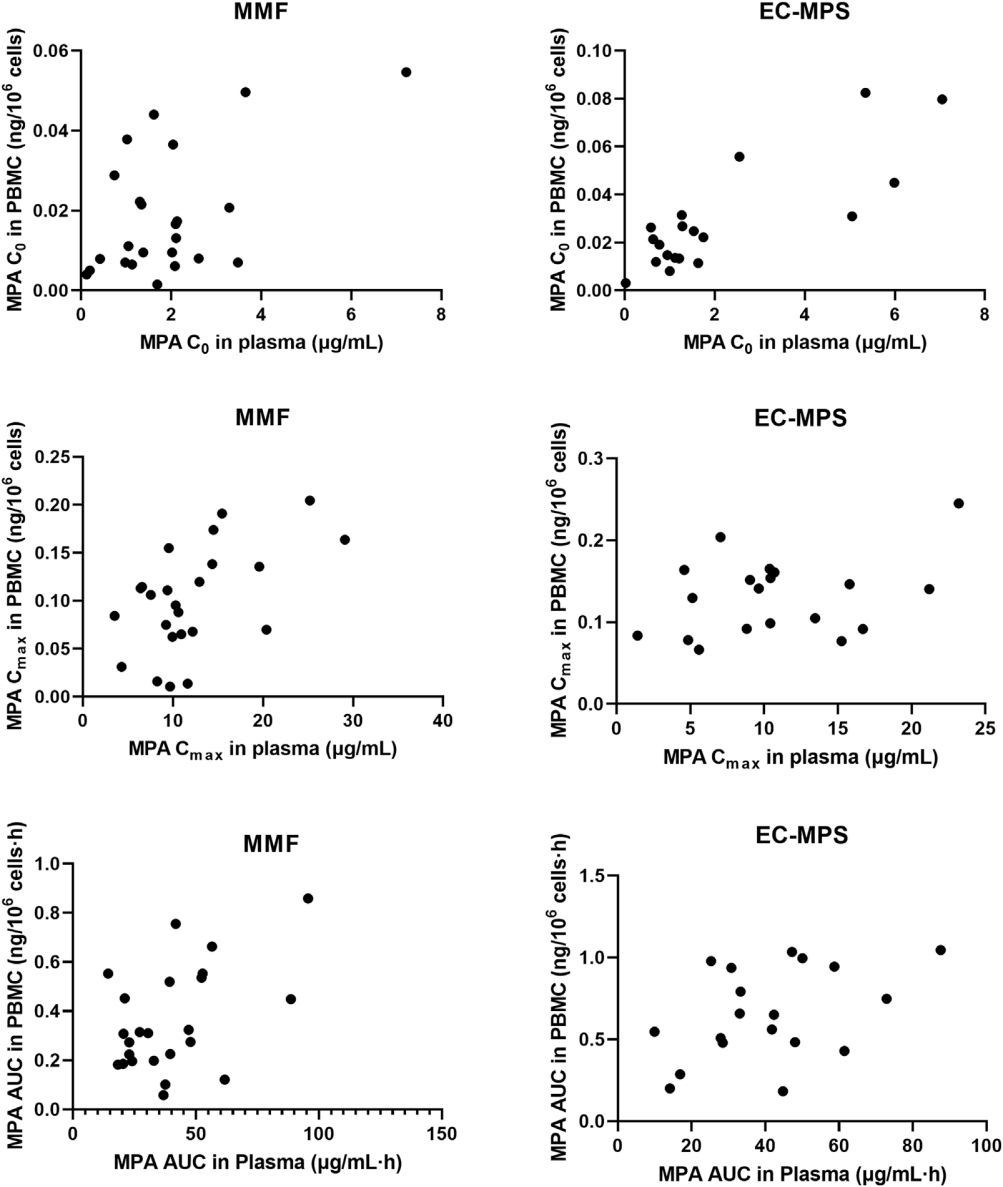
The relationship of C_0_, C_max_ and AUC_0-12_ of MPA in plasma and PBMC in Chinese renal allograft recipients after administration of MMF (n = 24) and EC-MPS (n = 19).

In patients receiving MMF, significant correlations were observed between plasma and PBMC C_0_, C_max_ and AUC_0-12_, with correlation coefficients of r = 0.535 (P = 0.007), r = 0.538 (P = 0.007), and r = 0.472 (P = 0.02), respectively. For patients receiving EC-MPS, the correlation of C_0_ was significant between plasma and PBMC (r = 0.821, P < 0.001). However, there was no statistical significant correlation of C_max_ (r = 0.348, P = 0.114) and AUC_0-12_ (r = 0.431, P = 0.065) (Figure 2).

### 3.3 IMPDH activity after administration of MPA

The IMPDH activity demonstrated a broad range for patients administered either MMF or EC-MPS (Table 2). The maximum inhibition (1-A_min_/A_max_) was 75.3% and 74.3% for patients administered MMF and EC-MPS, respectively.

**FIGURE 3:**
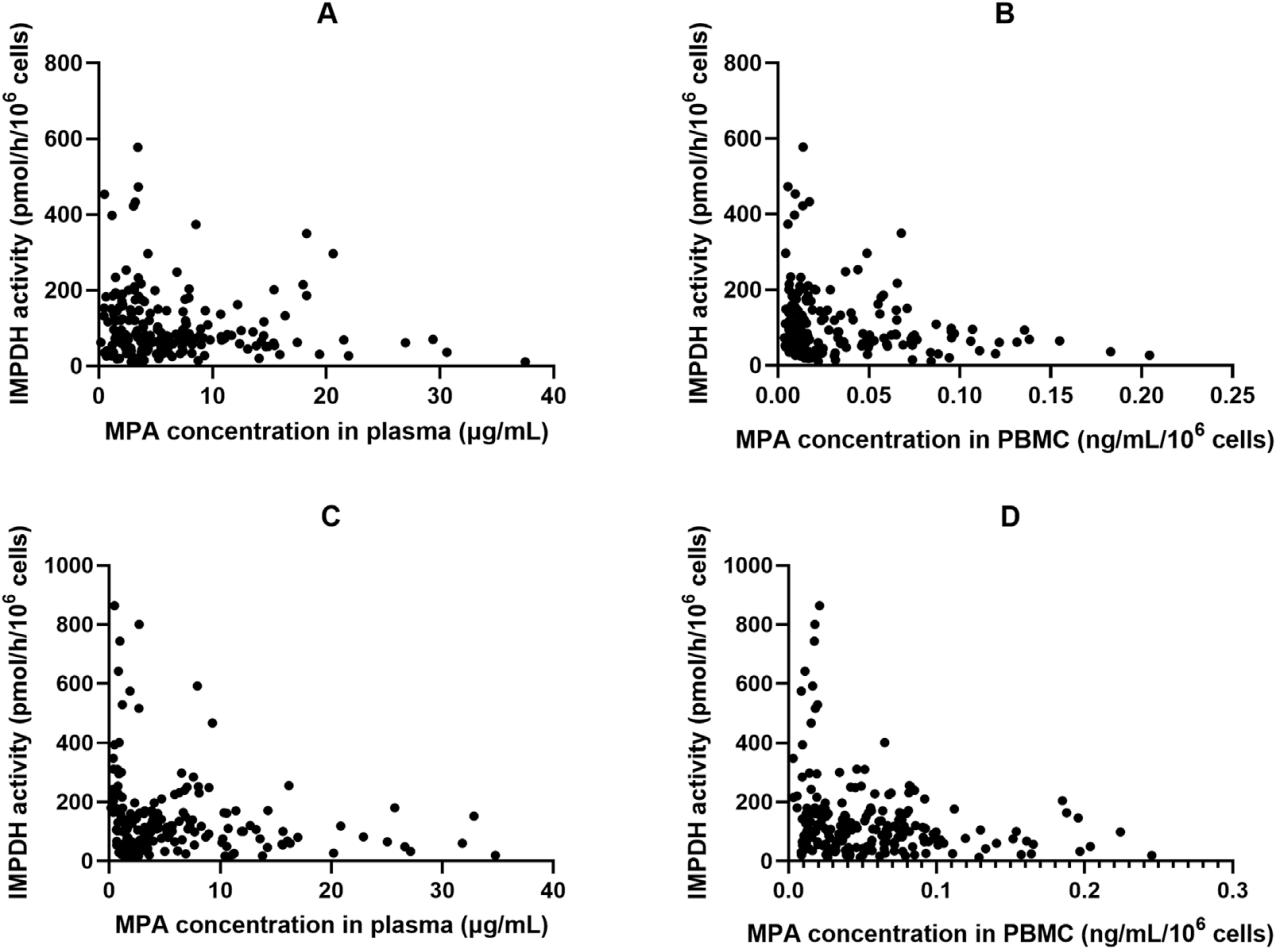
The relationship of inosine 5′-monophosphate dehydrogenase (IMPDH) activity and corresponding MPA plasma and peripheral blood mononuclear cells (PBMC) concentrations in Chinese kidney allograft recipients. **(A)** Plasma of patients administered MMF; **(B)** PBMC of patients administered MMF; **(C)** Plasma of patients administered EC-MPS; **(D)** PBMC of patients administered EC-MPS.

In patients receiving MMF, IMPDH activity attained its lowest level (67.9 ± 47.1 pmol·h^−1^·10^−6^ cells) at 1.5 h after dose administration, coinciding with peak MPA concentrations in both plasma and PBMCs (18.2 ± 9.28 μg·mL^−1^ and 0.10 ± 0.054 ng·10^–6^ cells, respectively; Figure 1). We found that 18 of the 24 patients exhibited A_min_ at 1.5 h post MPA administration. A similar pattern was observed in patients who received EC-MPS, though the MPA T_max_ in plasma and PBMCs of most patients were delayed up to 4 h, which in turn led to a corresponding delay in achieving maximum IMPDH inhibition. There were five patients each with A_min_ at 2 h, 4 h, and 6 h and three patients with A_min_ at 8 h after EC-MPS administration. Additionally, a correlation was observed between IMPDH AEC_0–12_ and MPA AUC_0–12_ in PBMC (r = 0.398, P = 0.01) (Figure 3).

### 3.4 PK-PD relationship in patients after MPA administration

**FIGURE 4:**
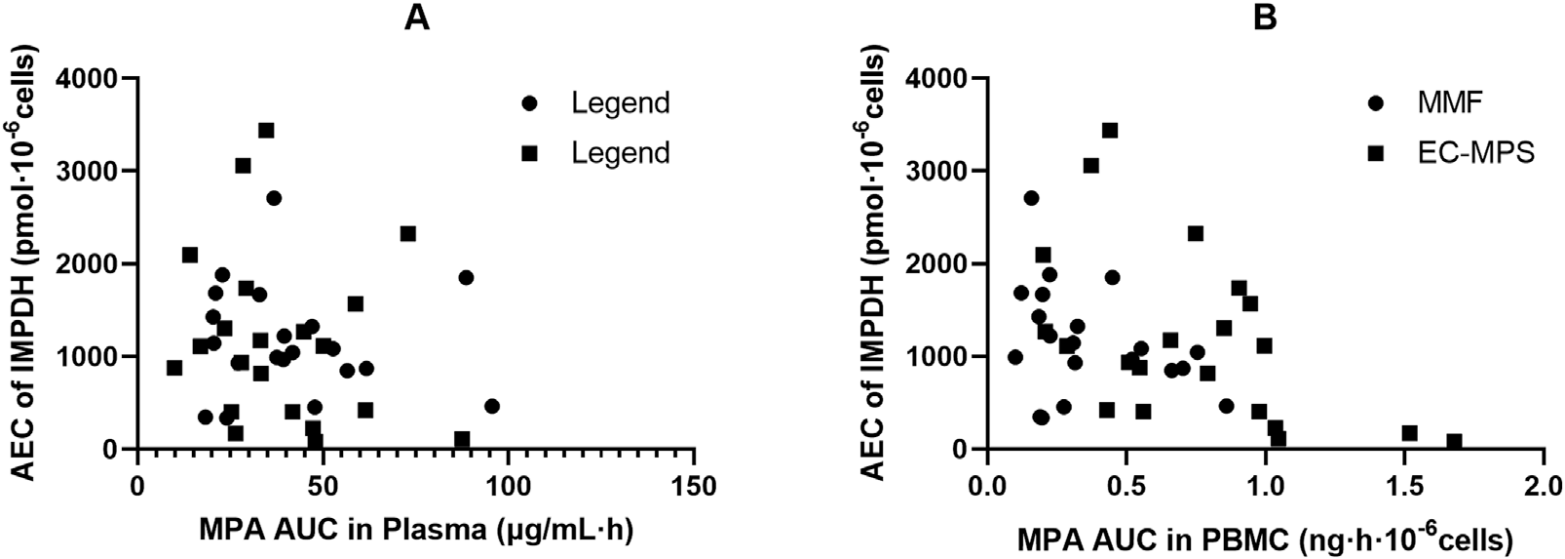
Association of AEC_0-12_ of inosine 5′-monophosphate dehydrogenase (IMPDH) and MPA AUC_0-12_ and in plasma **(A)** and peripheral blood mononuclear cells (PBMC) **(B)** of Chinese kidney allograft recipients.

Figure 4 shows the plot of IMPDH activity against MPA plasma concentration. The effect–concentration relationship between IMPDH activity and MPA after administration was evaluated. In both plasma and PBMCs, IMPDH activity generally decreased as MPA concentrations increased, thereby exhibiting an inverse relationship. The relationship between plasma MPA concentrations and IMPDH activity exhibited scattered data points, suggesting that MPA concentrations in PBMCs closely correlated with IMPDH activity.

An inhibitory sigmoid E_max_ model appropriately characterized the relationship between MPA concentration and IMPDH activity. The plasma MPA IC_50_ values were 2.76 and 3.34 μg/mL, whereas the PBMC MPA IC_50_ values were 0.023 and 0.052 ng/10^6^ cells, for patients administered MMF and EC-MPS, respectively. We found no significant differences in IC_50_ between patients administered MMF and those administered EC-MPS.

## 4 Discussion

In the present study, we comprehensively evaluated the relationship between IMPDH activity and MPA plasma or PBMC level for the first time in Chinese kidney allograft recipients during the early post-transplant period. We found a correlation of MPA PK between plasma and PBMC for patients receiving MMF and EC-MPS. Moreover, a comparable inhibition of IMPDH activity by MPA was found in patients administered MMF and EC-MPS.

The disposition of MPA *in vivo* is complex, with significant variability in absorption, metabolism, enterohepatic recycling (EHC), and renal excretion, which lead to considerable inter-individual differences in the PK of MPA. In our previous study, we found that various factors, including co-administered calcineurin inhibitors and genetic polymorphism of transporters, may impact the EHC. We also found that these factors further influence MPA exposure in Chinese allograft recipients (Sun et al., 2023). Intracellular MPA disposition may be a better PD index for immunosuppressive agents such as MPA (Capron et al., 2016; Sallustio, 2025). PBMCs are frequently used as the matrix in intracellular PK study, given that T-lymphocytes are important components of PBMCs. The relationship between MPA C_P_ and C_PBMC_ is modest and may be influenced by PBMC composition. In’t Veld et al. found that in healthy volunteers, MPA concentrations in T cells exceeded those in PBMCs (In ’t Veld et al., 2023). Nguyen et al. observed correlations between plasma and PBMC MPA concentrations at 1.5 and 3 h post-dose and a significant inverse relationship between IMPDH activity and both plasma and PBMC MPA concentrations (Nguyen et al., 2013). Md Dom et al. studied 48 kidney transplant patients who were administered MMF. They found that MPA C_0PBMC_ was weakly correlated with free MPA C_0_ (*r* ^2^ = 0.42, P = 0.013). MPA C_0PBMC_ were approximately 60% lower in recipients with rejection (P = 0.033). A significant concentration–effect relationship was suggested, with a threshold C_0,PBMC_ of 0.55 ng⋅10^–7^ cells providing 70% sensitivity and 67% specificity for predicting rejection (Md Dom et al., 2018). In another study, Riglet et al. built a three-compartment population PK model with a zero-order absorption and a first-order elimination, to describe MPA total and unbound plasma and MPA in PBMC of 78 adult kidney transplant recipients. They found CLcr has effect on CL/F of unbound MPA, serum albumin influences fraction of unbound MPA, and the *ABCB1 3435 C>T* genetic polymorphism has an effect on MPA efflux transport from PBMC (Riglet et al., 2020). In the present study, we compared MPA exposure in the plasma and PBMCs of Chinese kidney allograft recipients who received MMF or EC-MPS. A moderate correlation was observed between plasma and PBMCs for MPA C_0_, C_max_, and AUC_0–12h_ in patients administered MMF (*P* < 0.05; Figure 3). However, there is lack of a significant correlation between plasma and PBMC MPA exposure (C_max_ and AUC) in the EC-MPS group, contrary to the MMF group. We hypothesize that the EC-MPS, which leads to delayed and more variable absorption, may introduce complexity into the drug’s transfer into cells. This variability could obscure the correlation when using parameters like C_max_ and AUC. Future studies with more frequent sampling during the absorption phase are warranted to investigate this dynamic process further. Additionally, substantial inter-individual difference was observed in MPA PBMC concentrations. However, the fluctuation of MPA levels in PBMC was relatively stable compared with those in plasma (13.2 vs. 8.37 fold). This may be attributed to the equilibrium of efflux mediated by transporters on the cell membrane (Liu et al., 2023), which is a relatively slow process and results in more stable MPA exposure within PBMCs. For patients with extremely high or low MPA exposure, monitoring MPA concentrations in PBMCs may be clinically valuable.

The IMPDH inhibition by MPA and the subsequent nucleotide depletion causes further downstream consequences, including T cell proliferation and cytokine production (He et al., 2011; Pradier et al., 2019). IMPDH activity can be used as a surrogate index for monitoring MPA therapy (Vethe et al., 2014; Sobiak et al., 2020). Fukuda et al. (2011) demonstrated that high IMPDH activity before and after transplantation is associated with acute rejection. They observed that some kidney transplant recipients experience rejection despite achieving steady-state MPA concentrations, and these patients exhibit higher baseline IMPDH activity. Therefore, monitoring IMPDH activity may more directly reflect the degree of drug response and may help in preventing rejection and improving clinical outcomes. Pre-transplant IMPDH activity (Winnicki et al., 2022), pre-dose IMPDH activity (Thanukrishnan et al., 2022), or maximal IMPDH inhibition can be used to guide MPA dosing for improving outcomes and identifying patients at risk of acute rejection and MPA related side effects.

Consistent with previous studies, we found that in most patients, the highest inhibition of IMPDH activity was observed at the T_max_ of MPA. The observed degree of maximum IMPDH inhibition was comparable for kidney transplant patients receiving MMF and those receiving EC-MPS, with mean maximum inhibitions of 75.3% and 74.3%, respectively. The time of achieving A_min_ was 1–1.5 h for the MMF group and 2–6 h for the EC-MPS group. Additionally, A_min_ was observed at 8 h in two patients after MMF administration and in three patients after EC-MPS administration, an effect resulting from the EHC of MPA. No significant differences of AEC_0-12_ were observed among patients receiving MMF or EC-MPS (1163 ± 592 vs. 1146 ± 853 pmol·10^–6^ cells). We integrated the AEC_0-12_ of patients administered MMF and EC-MPS and found a correlation between AUC_0-12_ and AEC_0-12_ (Figure 3). MMF and EC-MPS seemed to exhibit similar effects on IMPDH activity in Chinese kidney allograft recipients. We used inhibitory E_max_ models to describe the overall relationship between the MPA concentrations and IMPDH activity in patients who received MMF and EC-MPS, respectively. We also estimated IC_50_ for patients, which reflects MPA concentration associated with inhibitory effect on IMPDH. The plasma IC_50_ values in patients who received MMF and EC-MPS were 2.76 and 3.34 μg/mL, respectively. The difference between the two values was not statistically significant. Fukuda et al. (Fukuda et al., 2011) reported an IC_50_ of about 1 mg/L in pediatric population. Tang et al. observed that the median IC_50_ values in young and older kidney transplant patients were 3.57 and 1.54 mg/L, respectively. The IC_50_ values in our study are comparable with those reported in previous studies (Tang et al., 2017). The discrepancy in IC_50_ values among various studies could be partly explained based on the differences in both the design of the studies (time points of sampling) as well as the determination method used.

According to previous study, the pharmacological effects of MPA are better described by the unbound MPA concentration (Sallustio, 2025; Smits et al., 2014). To the best of our knowledge, this is the first study that assessed the relationship between intracellular MPA exposure and IMPDH activity in Chinese kidney allograft recipients. We found that IMPDH activity exhibited an inverse trend with MPA concentrations in PBMCs, indicating a more significant correlation between PBMC MPA exposure and AEC_0-12_ than that between plasma MPA exposure and AEC_0-12_ (Figure 3). The MPA IC_50_ in PBMC was estimated as 0.023 and 0.052 ng · 10^–6^ cells by using the sigmoid E_max_ model, which were significantly lower than the value obtained from plasma MPA (2.76 and 3.34 μg/mL, respectively). Since the concentration in PBMCs directly reflects the pharmacological effect, and a weak correlation between PBMC and plasma MPA PK found in the present study, the PBMC concentration may serve as a stronger basis for IMPDH inhibition and PD in certain patients. However, further research into the PK-PD relationship between MPA in PBMCs and IMPDH activity will contribute to precise MPA therapy.

The present study has some limitations. First, the present study utilized a simple inhibitory E_max_ model to characterize the steady-state PK-PD relationship. A more dynamic, time-dependent model was not employed primarily due to limited data of PK and PD study of MMF and EC-MPS. Second, as all patients were recruited during the early post-transplantation stage, the study did not elucidate the relationships between PK of MPA and the clinical efficacy or adverse drug reactions. Third, while the therapeutic range for MPA exposure or IMPDH activity in PBMCs is valuable for therapy, as the present study was carried out in a single center, the number of patients met our inclusion criteria during the study period was limited, especially for patients administered with EC-MPS, we only estimated the relationship of MPA in plasma and PBMC. The therapeutic range should be established by using more data from different centers. Furthermore, future studies should explore the influence of genetic polymorphisms in drug transporters and metabolizing enzymes on the intracellular pharmacokinetics of MPA, which could provide further insights into the inter-individual variability observed.

## 5 Conclusion

In this study, we found a correlation between MPA exposure in PBMCs and plasma. The effects of MPA on IMPDH were similar for patients administered MMF or EC-MPS. The intracellular MPA concentrations exhibited a stronger correlation with IMPDH activity, suggesting that intracellular MPA levels, rather than plasma MPA levels, may better reflect the PD effects of MPA, under fully validation, it could lead to more personalized dosing regimens, particularly for patients experiencing efficacy failure or toxicity despite having plasma MPA concentrations within the target range.

## Data Availability

The original contributions presented in the study are included in the article/supplementary material, further inquiries can be directed to the corresponding authors.
